# Gut Microbiota Structure and Metabolites, Before and After Treatment in Early Rheumatoid Arthritis Patients: A Pilot Study

**DOI:** 10.3389/fmed.2022.921675

**Published:** 2022-07-08

**Authors:** Massimiliano Marazzato, Cristina Iannuccelli, Maria Paola Guzzo, Lucia Nencioni, Bruno Lucchino, Giulia Radocchia, Chiara Gioia, Giulia Bonfiglio, Bruna Neroni, Francesca Guerrieri, Fabrizio Pantanella, Stefania Garzoli, Marta Vomero, Cristiana Barbati, Manuela Di Franco, Serena Schippa

**Affiliations:** ^1^Department of Public Health and Infectious Diseases, Sapienza University of Rome, Rome, Italy; ^2^Early Arthritis Clinic, Department of Internal Medicine, Anesthesiology and Cardiovascular Sciences, Sapienza University of Rome, Rome, Italy; ^3^Department of Diagnostic Medicine and Radiology, UOC Clinical Pathology, Policlinico Umberto I, Rome, Italy; ^4^UMR INSERM U1052/CNRS 5286, Cancer Research Center of Lyon, Lyon, France; ^5^Department of Chemistry and Technology of Drug, Sapienza University of Rome, Rome, Italy

**Keywords:** rheumatoid arthritis, gut microbiota (GM), metabolomics, methotrexate, microbial network

## Abstract

Rheumatoid Arthritis (RA) is a chronic systemic autoimmune disease. Modifications of gut microbiota seem to be associated with the disease, but the impact of gut microbiota on therapies’ outcome remains unclear. A role of T cells in RA pathogenesis has been addressed, particularly on the Th17/Treg cells balance. Our study aimed to evaluate in early RA (ERA) patients compared to a control group, fecal gut microbiota composition, short-chain fatty acids concentrations, and the levels of circulating Th17/Treg and their own cytokines, before and after 3 months of standard treatment (Methotrexate (MTX) plus glucocorticoids). Fecal microbiota characterization was carried out on 19 ERA patients and 20 controls matched for sex and age. Significant decreased biodiversity levels, and a partition on the base of the microbiota composition, between the ERA patients at baseline compared to controls, were observed. The co-occurrent analysis of interactions revealed a characteristic clustered structure of the microbial network in controls that is lost in ERA patients where an altered connection between microbes and clinical parameters/metabolites has been reported. Microbial markers such as *Acetanaerobacterium elongatum*, Cristiansella massiliensis, and *Gracilibacter thermotolerans* resulted significantly enriched in control group while the species Blautia gnavus emerged to be more abundant in ERA patients. Our results showed an alteration in Th17/Treg balance with higher Th17 levels and lower Treg levels in ERA group respect to control at baseline, those data improved after therapy. Treatment administration and the achievement of a low disease activity/remission appear to exert a positive pressure on the structure of intestinal microbiota with the consequent restoration of biodiversity, of the structure of microbial network, and of the abundance of taxa that became closer to those presented by the subject without the disease. We also found an association between Blautia gnavus and ERA patients characterized by a significant reduction of propionic acid level. Furthermore significant differences highlighted at baseline among controls and ERA patients are no more evident after treatment. These data corroborate the role played by gut microbiota in the disease and suggest that therapy aimed to restore gut microbiota would improve treatment outcome.

## Introduction

The interaction between gut microbiota and autoimmune diseases is an intriguing and open topic.

The continuous improvement in Rheumatoid Arthritis (RA) knowledge is giving a great contribute to clarify pathogenic mechanisms of the disease with the consequent development of new therapeutic approaches. Despite the role of B lymphocytes in RA is well known, a crucial role of T cells has also been addressed and, in particular, the Th17 pathway is increasingly attracting the attention of scientific community. Th17 cells represent a dynamic subset of T-cells with a great plasticity that seems to play a crucial role in disease onset (1, 2). Generally, Th17 cells production is balanced with that of Treg cells and the main function of these last is to prevent autoimmunity by suppressing autoreactive lymphocytes. Treg cells can be divided in natural Treg cells, generated in the thymus in the early phases of life, and inducible Treg cells produced in the periphery during all life time. Treg cells exert their role immune suppressive role via cell-cell interaction or secretion of soluble molecules (such as IL-10 and TGF- β) (3). Because of their plasticity, Th17 cells differentiation is up-regulated in IL-6 microenvironment, while elevated level of TGF-β are able to induce Treg cells differentiation (4). Considering their opposite behavior and their reciprocal plasticity, a possible Th17/Treg cell imbalance in the pathogenesis of RA has been proposed. Moreover, several groups reported Il-17 as an important cytokine in the early phase of RA development, acting through the stimulation of other pro-inflammatory mediators and the induction of matrix metalloproteinases (MMPs) (5). Data from experimental models and human *in vitro* studies regarding RA, support a possible role of IL-17 in synovial tissue growth and in RANKL-independent osteoclastogenesis (6). The successful use of therapeutic inhibitors of these cytokines, corroborate their role in RA (7).

On the other side, a link between intestinal dysbiosis and autoimmune mechanisms involved in RA development has recently been shown (8). In particular, the involvement of gut microbiota in the pathogenesis of RA does not appear to be related to citrullinated proteins production, as for oral microbiota, but to the influence that colonizing bacteria have on cell-mediated responses. It has been demonstrated that intestinal bacteria are able to influence the balance between pro- and anti-inflammatory T cell subsets (9). In transgenic mouse model of inflammatory arthritis, gut microbiota has been shown to play a crucial role in the immune system development: germ free conditions determined a loss of Th17 cells in the small intestinal lamina propria While, after introducing a single gut-residing species, the lamina propria Th17 cells compartment and autoantibodies’ production were reinstated, with rapid arise of inflammatory arthritis (10).

Although in animal models of arthritis the role of gut microbiota has been established, it is still unclear how *dysbiosis* can influence RA onset and progression in humans. The first studies investigating the role of intestinal microbiota in humans were performed at the end of the last century using bacterial cultures of fecal samples, with the limit of the uncultivable bacteria. The presence of *Clostridium perfringens* was found to be more prevalent in RA patients compared to healthy subject and seemed to be associated with disease activity (11). In the years, several studies demonstrated that RA patients present intestinal dysbiosis and specific bacteria, such as Lactobacillus and *Prevotella copri*, are more abundant in untreated patients with recent onset of the disease (9). Later, using Next Generation Sequencing (NGS) techniques, lower levels of *Bifidobacteria* and bacteria of the *Bacteroides*-*Porphyromonas*-*Prevotella* group, *Bacteroides fragilis* subgroup, and *Eubacterium rectale* – *Clostridium coccoides* group, in fecal microbiota from early RA compared to controls were found. According to the available literature it seems to exist an association between a specific gut microbiota and RA, even if in some cases the results are in contrast, indicating that further investigations are necessary Moreover recent studies (8, 12, 13) indicate that gut microbiota has a strong influence on the individual response to medical therapies, while also contributing to drug metabolism. In order to provide a more specific causal attribution in the field of gut microbiota researches, a functional definition of dysbiosis, and not just a description of its composition, is indispensable. Among microbiota metabolites the Short-chain fatty acids (SCFAs) are involved in the promoting anti-inflammatory milieu, barrier homeostasis, and Treg cells (14). SCFAs are small organic acids with less than six carbons, produced by intestinal bacteria through the fermentation of the undigested components of food, mainly dietary fiber, in the large intestine (15). The maximum SCFAs concentrations are at cecum level, and the smallest in colon distal segments (16). Formic acid, acetic acid, propionic acid, butyric acid, isobutyric acid, valeric acid, and isovaleric acid encompass SCFAs list in mammalian (17, 18). Several gut bacteria secrete diverse quantities of SCFAs: Gram-negative bacteria, such as Bacteroides, mostly generate propionic acid and acetic acid; whereas Gram-positive bacteria, such as Firmicutes, mostly generate butyric acid (19). Among SCFAs, acetic acid, propionic acid and butyric acid have detailed physiological roles (20). Acetic acid owns anti-inflammatory activity and magnifies colonic blood flow and ileal motility (21, 22). Propionic acid also has anti-inflammatory activity (23). Propionic and acetic acids are both absorbed at gut level and passing through bloodstream circulation are able to reach and affect distant tissues. Conversely, butyric acid carries on its functions within the gut. Butyric acid provides a source of energy for intestinal cells and has a role in strengthening cell junctions, preserving the integrity of the colon epithelium and increasing mucin production (23–25). The present pilot study aimed to evaluate in Early Rheumatoid Arthritis (ERA) patients, before and after 3 months of standard treatment with Methotrexate (MTX) and glucocorticoids, and in a fibromyalgia patients group (as control), the fecal gut microbiota composition, the level of short-chain fatty acids (SCFAs) and of circulating Th17/Treg with their own cytokines. Furthermore by networks analysis, we highlighted the dynamic relations between microbes, SCFAs and patients clinical features.

## Materials and Methods

### Patient Population

We enrolled 25 consecutive Caucasian ERA patients fulfilling the ACR/EULAR 2010 classification criteria referring to the Early Arthritis Clinic of the Unit of Rheumatology – Internal Medicine, Anesthesiology and Cardiovascular Sciences, AOU Policlinico Umberto I, Rome. For all patients there was an indication to start therapy with traditional DMARDs according to the recent EULAR recommendations (26). Patients of both sexes, aged between 18 and 80, were included in the study only if they were ACPA and RF positive, with a disease duration less than 1 year and naive to any therapy. The exclusion criteria were: intake of antibiotic drugs within 60 days prior to sample collection, subjects with other inflammatory and autoimmune rheumatic diseases, diabetes mellitus, celiac disease or inflammatory bowel diseases, food intolerances, patients with contraindications to MTX therapy. All patients followed a Mediterranean diet.

As recommended by the current EULAR guidelines, all enrolled patients promptly started standard treatment with low doses of steroids (prednisone 5–10 mg/die or methylprednisolone 4–8 mg/die) and MTX (10 or 15 mg/week). The patients, maintaining a constant diet, have been evaluated at baseline (T0) and after 3 months of therapy (T1). At the first visit each patient underwent to a careful medical history, in particular relative to lifestyle, diet and drugs assumption. All enrolled patients underwent venous blood sampling, collecting of fecal sample, physical examination with tender and swollen joints count (TJC-28 and SJC-28) and clinimetric evaluation for the assessment of composite indices of disease activity (as DAS28, CDAI e SDAI) at T0 and T1. We also enrolled 20 control subjects, sex and age matched, who refer to the Rheumatology Clinic for other non-inflammatory rheumatologic diseases such as osteoarthritis or fibromyalgia. The controls underwent venous blood sampling and carried a stool sample only at baseline.

### Preparation of Peripheral Blood Mononuclear Cells and Flow Cytometry Analysis to Identify Th17 and Treg Cells

**FIGURE 1:**
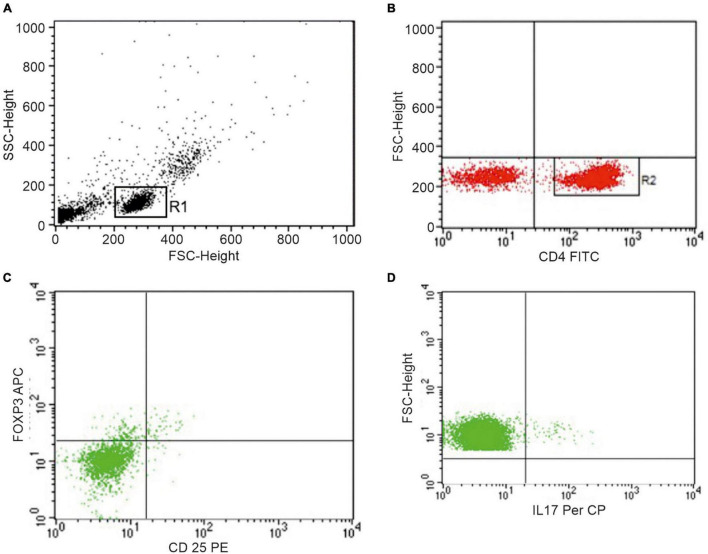
Representative dot plots of peripheral blood lymphocytes from one patient showing the gating strategy to identify Th17 and Treg cells. **(A)** Live cells, gated for cell death and debris exclusion, were then gated as lymphocytes (R1) based on morphological parameters (SSC, side scatter; FSC, forward scatter). **(B)** Identification of CD4+ lymphocytes (R2) within the R1 population. **(C)** Identification of Treg cells and **(D)** Th17 cells within the R2 population.

The peripheral blood mononuclear cells (PBMCs) were isolated by Ficoll-Hypaque density-gradient centrifugation to identify Th17 cells, defined as CD4 + T cells producing IL-17, and Treg cells, defined as CD4 + CD25 + FoxP3 + T cells. To avoid dead cells, surface and intracellular phenotyping of PBMCs was freshly performed with combinations of fluorescein isothiocyanate (FITC), phycoerythrin (PE), peridinin chlorophyll protein (PerCP) or allophycocyanin (APC)-labeled monoclonal antibodies (mAbs). For surface staining, after Fc-Receptor Blocking incubation, FITC conjugated mAbs against human CD4, and PE conjugated mAbs against human CD25 (all from BD Biosciences, San Jose, CA, United States) were used. The intracellular detection of IL-17/FoxP3 with PerCP-labeled anti-IL-17 (clone eBio64DEC17; eBioscience), APC-labeled anti-FoxP3 (clone 236A/E7; eBioscience) was obtained on cells fixed and permeabilized using Fix/Perm solution (eBioscience). For the detection of intracellular IL-17 production, PBMCs were stimulated for 4 h with 50 ng/ml phorbol 12-myristate 13-acetate (PMA) and 1 μg/ml ionomycin in the presence of 10 μg/ml Brefeldin A (all from Sigma-Aldrich, St. Louis, MO, United States), and then stained with PerCP-labeled anti-IL-17 mAb. For gating strategy of the analysis of Th17 and Treg cells, see Figure 1.

### Enzyme-Linked Immunosorbent Assay (ELISA) to Detect Serum Cytokines

Serum cytokine (IL-1β, TNF-α, IL-9, IL-23, IL-6, IL-17A, IL-21, IL-22, IL-10, and TGF-β) were assessed using a cytokine panel by Bio-Plex assay (Bio-Rad Laboratories, Richmond, CA, United States), following the manufacturer’s instructions. Data were analyzed using the Bio-Plex Manager software, version 4.1.1 (Bio-Rad Laboratories), reported as fluorescence intensity (FI) and subsequently converted into concentration (pg/ml).

### DNA Extraction From Fecal Samples

For the characterization of gut microbiota, fecal samples were collected from the ERA patients and control subjects enrolled in the study. The intake of antibiotic drugs in the 60 days preceding the collection of the samples was considered an exclusion criterion. From ERA patients we collected 2 stool samples, before (T0) and after 3 months (T1) of MTX therapy. A 200 mg of stool, from each sample collected, have been subjected to total DNA extraction with the Stool Mini kit (QIAGEN, Hilden, Germany), following the manufacturer’s instructions. In order to increase the DNA yield from Gram-positive bacteria, we modified the QIAGEN kit protocol by adding a further lysis step, a sample treatment with lysozyme (20 mg/ml) for 12 h in order to ameliorate bacterial walls lysis. The concentration of the extracted DNA was determined by Bio-Photometer^®^ spectrophotometer (Eppendorf, Milan, Italy) reading at 260 nm, as well as its quality with the 260/280 nm ratio. DNA integrity was checked through electrophoresis, in 1% agarose gel with 5 μl of SYBR™ Safe DNA Gel Stain. The characterization of the fecal bacterial community was carried out by Next Generation Sequencing (NGS), with Illumina MiSeq platform. To this purpose V3-V4 region of the bacterial 16S rRNA gene was amplified by PCR with specific universal bacterial primers using total DNA extracted as template. The library obtained was sequenced on Illumina MiSeq platform.

### Quality Control of the Sequences and Operational Taxonomic Unit Picking

**FIGURE 2:**
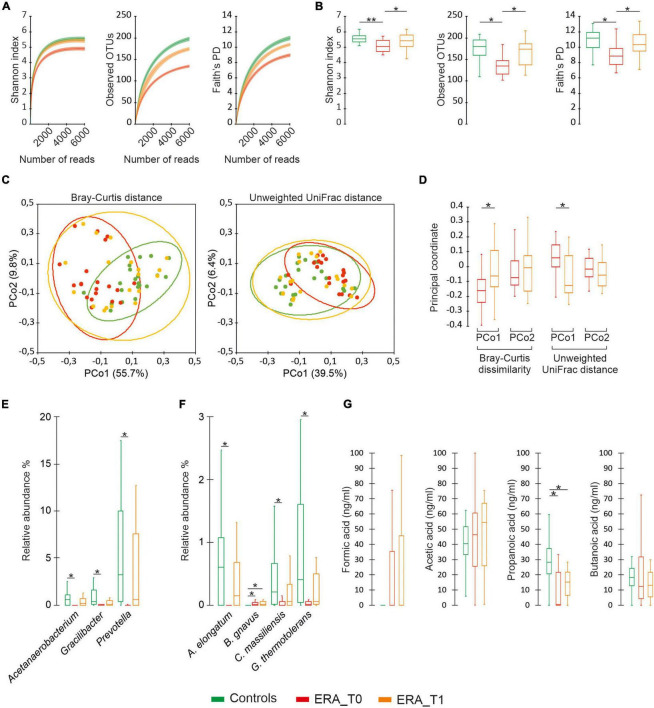
Microbiota diversity analysis. **(A)** Color coded rarefaction curves. For each group, the average values of α-diversity indexes with 95% confidence intervals were reported at different sequencing depth. **(B)** Color-coded box and whisker plots showing the distribution of the considered α-diversity estimators among groups. The presence of statistically significant difference between group of data were also reported. **(C)** PCoA plot of bacterial α-diversity based on Bray-Curtis dissimilarity and Unweighted UniFrac distance according to considered groups of subjects. For each group, the 95% confidence interval has been drawn. Numbers between parentheses represents the percentage of the total variance explained by the principal coordinates. **(D)** Color-coded box and whisker plots showing the distribution of principal coordinates for the ERA group between different time points. **(E,F)** Differential abundance analysis of taxa at genus and species levels with a mean relative abundance ≥1% determined in at least one group. **(G)** Distribution of SCFAs levels among groups. The Mann-Whitney U test and was performed to determine statistically significant differences between the control group and the RA one at both considered time-points. The Wilcoxon Signed-Ranks test was performed to assess significant differences between the different time-point considered for the ERA group. The * and ** difference is statistically significant.

After being demultiplexed reads were merged by USEARCH v11 (13) using a percentage identity threshold of 90% between aligned sequences. Raw data filtering for the presence of primers and low-quality sequences, as well as Operational taxonomic units (OTUs) identification were performed as previously described (27). In order to minimize artifacts, OTUs found in only one samples and/or presenting less than 10 sequences across the whole population were filtered out. Standardization of samples with unequal sequencing depths was performed by rarefacting to a total of 6,000 reads in agreement to rarefaction curves computed for each considered α diversity index (Figure 2). The Shannon index, Simpson index and number of observed OTUs were computed to evaluate α-diversity of each sample while the Bray-Curtis dissimilarity and the unweighted-UniFrac distances were used for β-diversity. PCoA was performed to compare the overall composition of the bacterial community within samples. The taxonomy assignment of OTUs was carried out by using a Naive Bayes classifier trained on a custom 97% clustered version of the Greengenes rDNA v13_10 in agreement with the method previously reported (14). A co-occurrence network was computed, at species level, for each group separately (Controls, ERA_T0 and ERA_T1), as previously described by Schippa et al. (28). Only bacterial species showing a mean relative abundance of 0.1% in almost one group have been considered for analysis. A second matrix based on Spearman rank sum correlation between the relative abundance of bacterial species and the clinical variables was computed for each group. Only values less than -0.6 and greater than 0.6 were considered for species-species correlations while values less than -0.5 and 0.5 were considered as thresholds for correlations between species and both clinical and metabolic variables. A *p* ≤ 0.05 based on bootstrapping of 1000 repetitions was considered as threshold to assess the statistical significance of considered correlations. For each group, the concurrence network was integrated with the Spearman rank sum correlations and, subsequently, data were imported and visualized in Cytoscape v. 3.7.2. Topological parameters relative only to the co-occurrence microbial networks were calculated by using the Network Analyzer plugin included in Cytoscape. Cluster of nodes highly connected by positive correlation has been determined by using the Glay algorithm included in clusterMaker plugin available for cytoscape (29). The presence of highly connected nodes (HUBs) were determined by using a degree-based approach considering both species-species and species-clinical variable interactions as previously reported (27).

### Analysis of Short-Chain Fatty Acids

To describe the SCFAs profile of the derivatized samples obtained we followed Tumanov et al. protocol (30), with some modifications, gas chromatograph (GC) coupled with a mass spectrometer GC system Clarus 500 model Perkin Elmer (Waltham, MA, United States) was used.

The GC was equipped with a Restek Stabilwax (fused-silica) polar capillary column and Helium was used as carrier gas at a flow rate of 1 mL/min. A 2 μL of each sample were injected in split mode (1:20) and the GC injector was set at 270°C. The temperature program was as follows: 35°C for 5 min and then increasing to 220°C at 10°C/min and finally held for 2 min. The mass spectra were recorded at 70 eV (EI) and were scanned in the range 29–300 m/z on source and the connection parts temperature was 230°C.

### Statistical Analysis of Data

A descriptive analysis of the samples was performed with tables and graphs on the base of the considered qualitative and quantitative variables. The chi-square test and the non-parametric Mann-Whitney *U* test were used to assess the presence of statistically significant differences between group at baseline respect to qualitative and quantitative variables, respectively. The Wilcoxon Signed-Ranks test was used to determine significant changes between the baseline and the considered endpoint. The per-mutational multiple analysis of variance (PERMANOVA) test with 1,000 permutations was calculated, on beta diversity distance matrices to assess the presence of statistically significant partitions between. Statistical calculations were performed by using XLstat software v 2016.02.28451 (Statsoft, United States) or R statistical environment version v3.2.5.^1^ In all cases, a *p*-value ≤ 0.05 was considered statistically significant. When necessary, the Benjamini–Hochberg false discovery rate (FDR) correction was used to account for multiple hypothesis testing.

## Results

### Clinical, Serological and Clinimetric Characteristics of Patient and Control Population

All patients were diagnosed with RA according the ACR/EULAR classification criteria (score ≥ 6) and the mean disease duration was 21 (±13.8) weeks. Patients who completed the follow-up were mostly females (*F* = 12, *M* = 7), as well as subjects in the control group (*F* = 19, *M* = 1); the average age was 55.8 (±13.8) and 49.3 (±12.8) years in patients and controls, respectively. Six patients drop out before completing the 3 months’ follow-up (intolerance to MTX, interruption of therapy for the appearance of respiratory infections). All patients were positive for ACPA and RF; in the control group, none of the subjects was ACPA or RF positive. As expected, both ESR and CRP were significantly higher in patients compared to controls (*p* < 0.0001; *p* < 0.0001, respectively). At T1, all serological, clinical and clinimetric parameters in ERA patients were significantly reduced. According to the DAS28 after 3 months of therapy (T0 DAS28 = 5.28 (±1.3) vs. T1 DAS28 = 2.7 (±1.2); *p* ≤ 0.0001) patients switched from a high disease activity to a low disease activity (Table 1).

**TABLE 1:** Demographic and clinical variables relative to the studied population of subjects.

	Controls No. 20	ERA T0 No. 19	ERA T1 No. 19	Significance
Sex male n (%)	1 (5%)	9 (45%)	–	^a^
Age (years) median (IQR)	53.5 (40.8–56.0)	61.0 (46.5–66.5)	–	
ACPA median (IQR)	0 (0–0)	320.0 (206.5–566.0)	92.0 (30.7–268.5)	^a,b^
RF median (IQR)	0 (0–0)	86.0 (23.5–237.5)	20.5 (0–59.2)	^a,b^
ESR median (IQR)	12.0 (8.0–19.2)	35.0 (21.5–52.5)	13.5 (6.0–20.0)	^a,b^
CRP median (IQR)	0.055 (0–0.2)	1.0 (0.41–1.45)	0.2 (0.07–0.37)	^a,b^
CDAI	–	21.5 (17.0–31.7)	3.5 (2.25–16)	^b^
SDAI	–	23.5 (18.0–37.5)	3.5 (2.25–17.875)	^b^
DAS28	–	5.5 (4.4–6.3)	2.3 (1.9–3.8)	^b^
CD4plus% median (IQR)	40 (31.5–45.2)	42.0 (38.0–45.8)	38.7 (34.2–42.6)	
Treg% median (IQR)	1.4 (1.1–1.8)	1.0 (0.8–1.3)	1.1 (1.0–1.8)	^a,b^
Th17% median (IQR)	0.85 (0.77–1.22)	1.8 (1.3–2.7)	1.5 (1.0–2.2)	^a,b^
IL9 median (IQR)	2.8 (0.7–3.2)	2.6 (1.9–11.9)	2.6 (1.0–5.4)	
TGFb median (IQR)	2513.4 (1782.7–3790.6)	1919.7 (1108.6–4185.5)	1903.4 (566.0–4103.7)	
TNF-alpha	–	3.3 (0–8.3)	0 (0–1.3)	^b^
IL6	–	35.1 (16.1–49.8)	13.6 (8.5–21.6)	^b^

*Statistical significance at alpha levels 0.05. ^a^: Controls vs. ERA T0, ^b^: ERA T0 vs. ERA T1.*

### Th17 and Treg Cells and Cytokines Levels

**FIGURE 3:**
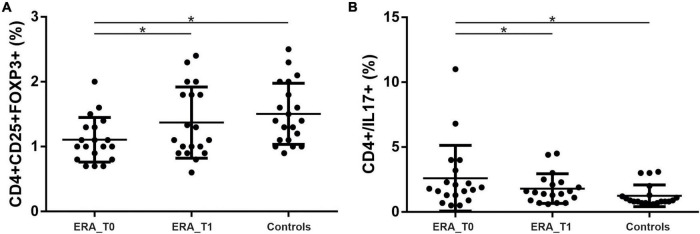
Percentage of Th17 and Treg cells in the peripheral blood of patients with rheumatoid arthritis at T0 and T1. **(A)** Significant increase in the percentage of Th17 in RA patients compared to controls and a reduction in the percentage of Th17 cells at T1 compared to T0. **(B)** Significant increase in the percentage of Treg cells in the control group compared to RA and a significant increase in Treg cells in RA patients at T1 compared to T0. * significant difference at alpha value 0.05.

**FIGURE 4:**
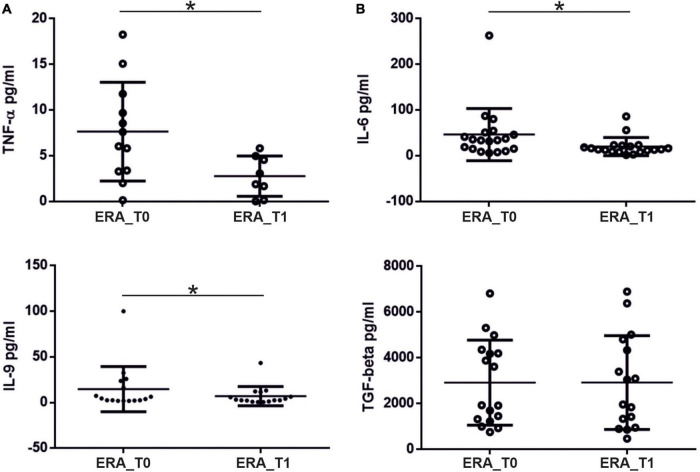
Levels (pg/ml) of pro-inflammatory cytokines measured in the serum of patients with RA at T0 and T1. **(A)** Significant reduction in serum TNF-α levels and **(B)** IL-6 levels at T1 compared to T0 in patients with rheumatoid arthritis. * significant difference at alpha value 0.05.

We investigated the percentage of Th17 and Treg cells in the peripheral blood of patients with RA at baseline (T0) and after 3 months of therapy (T1). As shown in Figure 3 at baseline, Th17 + cells were significantly higher in the peripheral blood from RA patients compared to controls (*p* = 0.0139); at T1, Th17 + cells were significantly reduced in RA patients (*p* = 0.02). At baseline, we also found that Treg cells were significantly higher in the control group compared to RA patients (*p* = 0.0048); at T1, a significant increase in Treg cells in RA patients was observed (*p* = 0.0014). A positive correlation between the percentage of circulating Th17 cells and disease activity, calculated by both DAS28-VES and DAS28-CRP, was found (*p* = 0.0353, *r* = 0.3425 and *p* = 0.0497, *r* = 0.320, respectively). Regarding circulating levels of different pro-inflammatory and immunoregulatory cytokines evaluated both at T0 and at T1, we only found detectable levels of TNF-α and IL-6. Specifically, at T1we found a significant reduction of both TNF-α (*p* = 0.0313) and IL-6 (*p* = 0.0124) serum levels compared to T0 in ERA patients (Figure 4).

### Early Rheumatoid Arthritis Patients-Associated Dysbiosis Is Restored by Treatment

The microbiota analysis determined 545 different OTUs, which have been assigned to 247 species and 130 different genera. The generated OTUs have been used as the basis for comparative analysis between groups with respect to ecological measures of diversity. As showed in Figure 1, the control group revealed significantly higher values of alpha-diversity compared to ERA patients at baseline, compared to the ecological indexes considered. No significant differences were observed between the control group and the ERA one at T1. Beta-diversity analysis evidenced statistically significant partitions between controls and the ERA group at baseline (Bray-curtis *p* = 0.003; unweighted UniFrac *p* = 0.006), while no significant separation was displayed between controls and ERA subjects at T1. The presence of a significant separation between ERA patients at baseline and after the MTX treatment, has also been evidenced by the application of paired Wilcoxon sign rank test on the first main coordinate (Figure 2D). Overall results indicate that: (1) a specific composition of gut microbiota seems to be associated to ERA pathology; (2) MTX treatment seems to bring the microbial composition back to that characteristic of not diseased subjects. The evaluation of taxa relative abundance evidenced statistically significant differences among studied groups. At genus level, *Acetanaerobacterium*, *Gracilibacter*, and *Prevotella* presented significantly higher values in control subjects compared to ERA patients at baseline. Significant differences in the microbiota composition disappear when control group was compared to the ERA one at T1. Similar results were observed at the species level for *Acetanaerobacterium elongatum*, *Cellulomonas massiliensis*, and *Gracilibacter thermotolerans*. All three species were found to be significantly more abundant in control group compared to ERA patients at baseline while no significant differences were observed by comparing controls with the ERA group at endpoint. Notably, *Blautia gnavus* was the only taxa maintaining significantly higher relative abundance in the ERA patients at endpoint compared to controls (Figure 2F).

### Analysis of Short-Chain Fatty Acids Reveals Significant Differences Among Control and Early Rheumatoid Arthritis Patients

The metabolomic analysis of SCFAs showed that ERA patients presented significantly decreased propanoic acid levels at baseline compared to controls. Such differences persisted after MTX administration to the ERA group although the tendency to increase observed for such compound would suggest the need for a prolonged duration of treatment for its complete restoration to normal level (Figure 2G). No significant differences were found for formic acid, acetic acid, and butanoic acid assayed showing that quantitative variations of this SCAFs are not associated with ERA or modified by the treatment with MTX.

### Co-occurrence Analysis Reveals Definite Gut Clustered Networks Altered by Rheumatoid Arthritis

**FIGURE 5:**
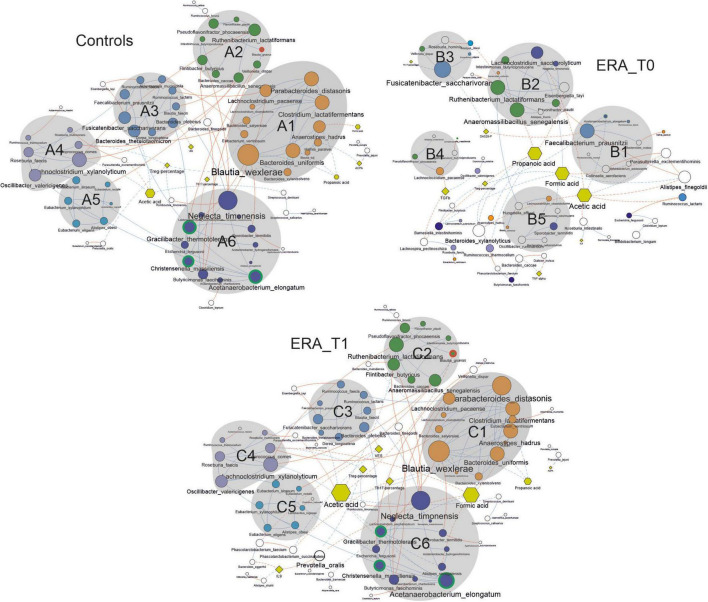
Co-occurrence network analysis taking into account bacterial species presenting a mean relative abundance ≥ 0.01% across the whole population of samples. Node size is proportional to the number of edges departing from the node, while the edge thickness is proportional to the strength of correlations. Node name size is proportional to the betweenness centrality, meaning the bridging/key importance of that node within the network. Different shapes are used for nodes representing species (circles), clinical variables (rhombus) and metabolic variables (exagons). Continuous lines represent correlations between species while dotted ones indicate correlations between species and clinical and metabolic variables. Blue and red edges represent positive and negative correlation, respectively. Nodes are colored in agreement with the modularity class detected by the GLay algorithm in the network relative to the control group.

A methodology to study the function and assembly of the microbiota ecosystem is to describe the microbial interactions. We constructed a graphical representation of microbial networks for each group/time-point separately (Figure 5). Such network reported the co-occurrence/co-exclusion of species, as well as, their correlations with meaningful clinical variables and microbial metabolites. For each computed co-occurrence network, topological parameters have been determined. Obtained results evidenced a more connected network in control subjects compared to the ERA group at baseline, differences that did not survive in the network of ERA group at T1 (Table 2). The modification of microbial networks, in terms of participants (nodes), number of interactions (edges) and connectivity, was clearly restored in ERA patients after MTX therapy.

**TABLE 2:** Topological parameters of co-occurrence networks computed for each group/time-point.

	CTRL	ERA-T0	ERA-T1
Nodes	70	58	81
Edges	102	57	105
Edges/node ratio	1.45	0.98	1.29
Synergisms	77	47	75
Exclusions	25	10	30
Syn/Escl ratio	3.08	4.7	2.5
Average number of neighbors	3.11	2.3	2.96

The graphical representation of microbial interactions showed that control group presented a higher number of highly connected modules (groups containing ≥ 4 nodes) compared to those showed by the ERA group at baseline (T0). Modules determined for the control group tended to comprise higher number of nodes, than that observed in the ERA group before therapy (Figure 5). Within the co-occurrence network computed for the control group, the module identified as A6 comprised the three bacterial species resulted to be significantly enriched in this group. The analysis of highly connected nodes (hubs), underlining the keystoneness of species, evidenced that all three taxa play a major role in gut ecosystem (Supplementary Table 1). The module A6 resulted to be positively correlated with other three clusters of bacterial species (A3, A4, and A5) including bacterial taxa such as *Faecalibacterium prausnitzii*, *Bacteroides thetaiotaomicron*, *Akkermansia muciniphila*, and *Roseburia faecis*. *F. prausnitzii*, and *B. thetaiotaomicron* constituted major hubs in the network, while all the four species have been previously associated with human health status. Furthermore, the A6 module resulted to be negatively correlated to ESR values, although indirectly, through the species *Bacteroides plebeius*. This module, together with the A3, A4 and A5 ones, resulted to be inversely correlated with the A2 cluster of taxa containing the *B. gnavus* species, significantly enriched in ERA patients, and the biggest module A1, positively correlated with the ACPA levels. The latter modules included bacterial taxa previously associated with gut dysbiosis in different pathological contexts such as *Parabacteroides distasonis*, *Eubacterium ventriosum*, and *Anaerostipes hadrus* (31–35).

The co-occurrence network determined for ERA patients at baseline evidenced several small groups of species appearing to be isolated from the rest of the community. Notably, the major clusters determined in this microbial network are composed of bacterial species included in different clusters within the network relative to control subjects. This finding highlights that ERA is associated with changes in the structure of microbial network within the human gut ecosystem. Interestingly, in the ERA network almost all the hub species found in controls seemed to lose their keystoneness while others, comprising *F. prausnitzii*, resulted to assume a major role.

Similarly to the network computed for the control group, the one determined for the ERA at baseline showed that modules containing species with a key role for eubiosis or resulting significantly more abundant in the control group (e.g*., A. elongatum*) were negatively correlated with the clinical variables used to evaluate ERA severity, such as DAS28-PCR. Furthermore, it is interesting to note that the microbial network determined for the ERA group after MTX treatment (T1) was characterized by restoration of quite the same clusters of species observed in controls and by similar interaction between these clusters and the clinical variables assessing disease severity. These results strongly suggest that, within the gut, the eubiotic state is associated with peculiar pattern of microbial interactions whose perturbation could be directly associated with the onset of RA. Concerning metabolic variables, the analysis of main nodes within networks revealed that, in ERA subjects, acetic acid, formic acid and propionic acid represent key metabolites while their role result to be significantly resized in controls and ERA patients after treatment, except for formic acid.

A positive correlation between Treg percentage and *Coprococcus comes* has been determined in the networks relative to controls and ERA patients after therapy. The Treg percentage and the Th17 levels were negatively correlated with *Neglecta timonensis*, in both the networks relative to control group and ERA patients at baseline.

The propionic acid resulted to be significantly decreased in ERA patients at T0 and T1, and it resulted to be positively correlated with several species belonging to the genera *Blautia*, particularly with *Blautia wexlerae*, in the networks computed for control group and ERA patients at T1. The bacterial species *B. wexlerae*, *C. comes*, and *N. timonensis* were all included in specific sub-cluster within the network relative to controls and in that concerning ERA patients at T1. In addition, all three species strongly connected with other taxa, delineating them as keystone species. In ERA patients at T0 network the species mentioned above are present, but their connections are reduced and their keystoneness is no longer supported. Interestingly, *B. gnavus*, the only species with relative abundance significantly higher in both ERA groups (before and after MTX treatment) compared to control, is detectable only in the networks of controls and ERA at T1.

## Discussion

The study aimed to characterize fecal gut microbiota and fecal SCFAs concentrations in ERA patients compared to controls and to analyze their variation after 3 months (T1) of MTX plus glucocorticoids treatment in ERA patients, along with the estimation of clinical and clinimetric features. At the same time, we evaluated the relationship between circulating Th17/Treg levels, their own cytokines and gut microbiota by network analysis.

Our results showed significant differences in all the ecological index considered to evaluate alpha and beta diversity comprising those integrating genetic distances among groups of microbes. Obtained data strongly evidenced that not only untreated ERA patients are characterized by a lower microbial biodiversity compared to controls, but also that, on the base of the global gut microbial composition, it is possible to determine a significant partition between the two groups, evidencing a wide involvement of microbiota structure in the modification associated with ERA. The significant differences found in biodiversity, global microbiota composition and the relative abundances of specific genera and species between controls and untreated ERA patients disappeared after administration of therapy, indicating a plausible positive pressure exerted by the treatment on gut microbiota structure. Our results corroborate already reported findings, describing dysbiosis in RA patients or changes in gut microbiota of RA patients after therapy (36–38), even if several differences in the studies design could be evidenced. Differently from our results, in Picchianti Diamanti study (36) the gut microbiota composition has been analyzed in three different groups of RA patients, a group of patients treatment-naïve, one group treated with methotrexate and etanercept, and a group treated only with etanercept (ETN). Zhang and collaborators analyzed oral and fecal microbiota of 77 patients with RA, naïve to therapy (38). The authors evidenced the presence of gut dysbiosis in ERA subjects, as well as, a partial restoration of microbiota after treatment with DMARDs comprising MTX treatment. Although these findings are in agreement with those evidenced by our study, the underlined modifications in charge to the gut microbiota were mostly evidenced by analyzing assemblages of genes, or a limited number of specific microbial markers. In this context it is important to underline that such results, although meaningful, are not able to evidence that ERA associated gut-dysbiosis involved the whole composition of gut microbiota and/or the structure of the microbial network of interaction within the gut ecosystem. We are able to provide additional information about the clustered structure of gut microbial community, as well as its modifications in ERA patients treated with glucocorticoids and MTX. Furthermore, we highlighted the dynamic governing relationships among microbes, SCFAs and clinical features in the microbial networks. We observed an increase in the genera *Acetanaerobacterium* and *Gracilibacter*, both belonging to the Firmicutes phylum and the Clostridiaceae family, that seems to be due to an expansion of the species *A. elongatum* and *G. thermotolerans*. No data are reported regarding these species in RA or other pathologies. Previously Maeda and colleagues reported *P. copri* as a gut microbe associated with patients with early RA, who did not receive any treatment (39–41). Our results indicate a significantly higher abundance of Prevotella genera in ERA group at baseline, when compared to control group, but no significant differences regarding *P. copri* were shown in the groups studied. However, data reported in literature on *P. copri* pathogenicity in patients with RA are still discordant (40, 42). It could be possible that the functional role evidenced for *Prevotella* spp. in our data is linked to the genera rather than to a single species within this bacterial group. The species *B. gnavus* (a species named before *Ruminococcus gnavus*, that have been reclassified as *Blautia*) resulted to be significantly higher in ERA patients at baseline (T0) and follow-up (T1), compared to control group. *B. gnavus* is a predominant microbe living in the gut, found in 90% of people. In healthy human subjects, *B. gnavus* represents < 0.1% of the microbiota (43). *B. gnavus* is a mucosa associated microbe, which colonize the intestinal mucosa surface, and utilize the sialic acid from mucin glycans as carbon source (44). Our study is the first one that associates this bacterial species with ERA patients. The relative abundance of *B. gnavus* has been found increased in Crohn’s Disease (CD) patients compared to healthy individuals (45). The increases in *B. gnavus* have been linked to other inflammatory diseases such as spondyloarthritis (46), pouchitis in ulcerative colitis patients who have experienced a total colectomy (47), and eczema in children (48). In order to add a comment we have improved the discussion section with the sentence. In a study by Breban et al. (46) a significantly greater presence of *B. gnavus* in patients with spondyloarthritis, compared to patients with RA, is reported. In our study, comparing patients with RA and our controls (patients with non-inflammatory rheumatologic diseases, such as fibromyalgia), we found significant greater presence of *B. gnavus* in RA patients, indicating that the extent of inflammation is a factor able to influence its presence. *B. gnavus*’s ability to use mucin as a carbon source (44, 49) could directly interfere with gut barrier function, leading to an increased colonization of the mucin layer that could rise the immune system’s exposure to inflammatory triggers factors. Nevertheless, we have to say that the species of the genus *Blautia*, a Gram negative anaerobic bacteria, have also been indicated as bacteria with probiotic characteristics (50). Results obtained on fecal SCFAs analysis showed a significant decrease of propionic acid in fecal samples of ERA patients at T0 and T1 compared to controls. Propionic acid is well-known in the literature for its ability to improve insulin sensitivity, promote the synthesis of neurotransmitters, as well as act on lipid metabolism (51, 52), and immune system (53). Propionic acid has been shown to be able to determine, in the intestine, a significant increase of Treg population in a mouse model of Parkinson’s disease (54). A recent work showed how propionic acid determines a greater expression of genes favoring Treg proliferation in the gut (55, 56). Interestingly, in ERA patients at T1, we found that propionic acid is positively correlated, although indirectly, with Treg, while a negative and direct correlation linked the two variables in the same patients before the MTX therapy, indicating changes in the interactions between host immunity/bioactive molecule/healthy status. Further studies will be necessary to better comprehend those modifications and their connections with the pathology. Th17 parameter was positively correlated, in controls and ERA patients at T1, with the obesogenic species *E. ventriosum*, able to improve energy harvesting from the diet (33). The metabolite propionic acid was the only metabolite resulted to be significantly decreased in ERA patients, both T0 and T1 groups, compared to controls. Regarding propionic acid, only in the networks of controls and ERA patients at T1, we observed positive correlations with species belonging to the genus *Blautia*, in particular with the species *B. wexlerae* and *B. luti*. Species belonging to the *Blautia* genus appear to be strongly connected, indicating their potential key role in the fecal microbial ecosystem of these taxa. As reported before, the genus *Blautia* encompasses anaerobic bacteria with probiotic characteristics, widely present in mammalian feces and intestines (50). Interestingly, *B. gnavus* is only demonstrable in the networks of control subjects and ERA patients after therapy. It would seem that, at the beginning of the pathology, even if present in greater amounts than in control group, *B. gnavus* does not play an active role in the balance of microbial ecosystem, in fact it is not connected with other taxa and is not present in any existing clusters in the network. It could be that the intestinal environment before treatment, even appearing to favor its growth, does not allow its interaction with other species, which instead occurs after treatment, and in the absence of RA disease. Moreover, our results showed that the expression of Th17 cells is greater in patients than controls and decreases significantly after therapy going hand in hand with clinical response whereas, on the contrary, Treg cells are more expressed in controls that do not have a systemic inflammatory disease and, in the group of patients, they increase significantly after therapy. The values of Th17 and Treg cells in ERA patients after pharmacological therapy are comparable to those of controls. All ERA patients have been treated with low dose of glucocorticoids plus MTX considered the standard treatment according to international guidelines (57) and the most of them switched from a high/moderate disease activity to a low disease activity/remission as expected. It is well-known that early treatment of RA leads to good therapeutic results as previously described in literature (58, 59). Our results suggest that the achievement of low disease activity or clinical remission in ERA treated subjects leads to a positive impact on gut community structure and of Th17 and Treg levels. This effect, that seems to be therapy-induced, is probably independent from a specific drug but could be due to the reduction of inflammation levels. This hypothesis is corroborated by the significant relation between Th17/Treg levels and disease activity. It is therefore possible to hypothesize that these results are correlated with clinical response and, for this reason, traceable also in patients treated with other drugs. In ERA patients at T0 Th17 + cells were significantly higher respect to controls, and reduction at T1 compared to T0 was observed. On the contrary, Treg cells resulted to be significantly higher in the control group compared to ERA patients at T0 and a significant increase was observed in ERA patients at T1 compared to T0. We found a positive correlation between the percentage of circulating Th17 cells and disease activity, calculated by both DAS28-ESR and DAS28-CRP. After MTX and glucocorticoids treatment, as expected we observed a significant reduction of TNF-α and IL-6 levels, indicating an impact on the immune responses, and corroborating the Treg/Th17 balance variation observed in ERA patients between T0 and T1. Finally, network analysis showed a positive correlation between Treg percentage and *Coprococcus comes* in controls and ERA patients after therapy. Furthermore, Treg and Th17 were negatively correlated with *Neglecta timonensis*, in control and ERA patients at T1. Results indicate a partial restoration of the relations among immune cells and bacteria after therapy. Our study has been conducted in a group of patients suffering from RA of recent onset and naive to any drug. The small sample size is a limitation of the study but, at the same time, we made a careful selection of patients excluding those who had taken any drug, selecting subjects with recent onset and homogeneous from the serological point of view for the presence of ACPA and RF. We report for the first time an association between the bacterial species *B. gnavus* and ERA patients. The achievement of low disease activity or clinical remission thanks to therapy induces changes in gut microbiota of patients with ERA. In this context, ERA patients after treatment showed a more connected network, restoring the interactions patterns of controls These results confirm those obtained in the evaluation of α and β diversity. Some studies showed an antibacterial activity of MTX (60, 61). Both of these studies were conducted *in vitro*, a different situation from that occurring *in vivo*. *In vivo* microbes live in complex ecosystems, assuming metabolic characteristics very different from those observed *in vitro* in monoculture. From our data it is not possible to say whether MTX has direct effects on microbiota (mediated by its antibacterial activity) or if the effect is mediated indirectly by the reduction of inflammation. It is possible that both actions concur to the final effect observed. Further studies will be necessary to clarify this point. We should finally mention the study by Isaac et al. (62) reporting that oral MTX therapy alone did not produced major changes in the fecal microbiota structure, in contrast with our results. The research has been conducted on two groups of patients with early RA: the first group received vancomycin followed by MTX, the second one received only MTX. In this study an older sequencing platform (Roche 454 FLX) characterized by less sequencing depth, as well as more limited quality of called basis, was used to characterize gut microbiota. Moreover, the applied statistical methods employed were different. Both factors could strongly impact on final results. In conclusion, our results seem to indicate a positive impact of therapy on gut community structure, we underline significant partition between controls and ERA patients as well as the restorative effect exerted by MTX treatment, considering the whole microbial composition. We further confirmed this evidence by underlining a clustered structure for microbial relationship that revealed to be altered by ERA while restored by treatment. A possible improvement in the management of RA patients could be achieved by therapies aimed at restoring microbiota ecosystem, in support of traditional therapies.

## Data Availability Statement

The original contributions presented in this study are publicly available. This data can be found here: NCBI SRA repository, accession: PRJNA832901.

## Ethics Statement

The studies involving human participants were reviewed and approved by the Bioethics Committee of the Sapienza University of Rome. The patients/participants provided their written informed consent to participate in this study.

## Author Contributions

MM performed the bioinformatic and statistical analysis and contributing to writing the first draft. CI contributed to the study design and writing the first draft. MG contributed to the conception of the study and enrolled the patients. BL and CG contributed to the enrollment of the patients. FG was responsible for the sequencing process. SG performed the metabolomic analyses. GR, GB, and BN contributed to sample processing. FP and LN contributed to protocols design and sample processing supervising. MV and CB performed PBMCs isolation, flow cytometry experiments, and detection of cytokines. SS and MD contributed to the conception and design of the study and wrote the first draft of the manuscript. All authors contributed to manuscript revision, read, and approved the submitted version.

## Conflict of Interest

The authors declare that the research was conducted in the absence of any commercial or financial relationships that could be construed as a potential conflict of interest.

## Publisher’s Note

All claims expressed in this article are solely those of the authors and do not necessarily represent those of their affiliated organizations, or those of the publisher, the editors and the reviewers. Any product that may be evaluated in this article, or claim that may be made by its manufacturer, is not guaranteed or endorsed by the publisher.

## Footnotes

1
https://cran.r-project.org
